# Dietary use and conservation concern of edible wetland plants at indo-burma hotspot: a case study from northeast India

**DOI:** 10.1186/1746-4269-7-29

**Published:** 2011-10-04

**Authors:** A Jain, M Sundriyal, S Roshnibala, R Kotoky, PB Kanjilal, HB Singh, RC Sundriyal

**Affiliations:** 1 North-East Institute of Science and Technology (CSIR), Substation, Imphal 795004, India; 2 G.B. Pant Institute of Himalayan Environment & Development, Kosi-Katarmal, Almora, Uttarakhand-263643, India; 3 North-East Institute of Science and Technology (CSIR), Jorhat 785 006, India

**Keywords:** Wetland plant resources, tribal communities, dietary use, ethnobotanical survey, livelihood, marketing, nutritive value, conservation

## Abstract

**Background:**

The wetlands of the North East India fall among the global hotspots of biodiversity. However, they have received very little attention with relation to their intrinsic values to human kind; therefore their conservation is hardly addressed. These wetlands are critical for the sustenance of the tribal communities.

**Methods:**

Field research was conducted during 2003 to 2006 in seven major wetlands of four districts of Manipur state, Northeast India (viz. Imphal-East, Imphal-West, Thoubal, and Bishnupur). A total of 224 wetland-plant-collectors were interviewed for the use and economics of species using semi-structured questionnaires and interview schedules. Imphal, Bishenpur and Thoubal markets were investigated in detail for influx and consumption pattern of these plants. The collectors were also inquired for medicinal use of wetland species. Nutritive values of 21 species were analyzed in laboratory. The vouchers were collected for all the species and deposited in the CSIR-NEIST (*Formerly Regional Research Laboratory*), Substation, Lamphelpat, Imphal, Manipur, India.

**Results:**

We recorded 51 edible wetland species used by indigenous people for food and medicinal purposes. Thirty eight species had high medicinal values and used in the traditional system to treat over 22 diseases. At least 27 species were traded in three markets studied (i.e. Imphal, Thoubal and Bishenpur), involving an annual turnover of 113 tons of wetland edible plants and a gross revenue of Rs. 907, 770/- (US$1 = Rs. 45/-). The Imphal market alone supplies 60% of the total business. Eighty per cent of the above mentioned species are very often used by the community. The community has a general opinion that the availability of 45% species has depleted in recent times, 15 species need consideration for conservation while another 7 species deserved immediate protection measures. The nutrient analysis showed that these species contribute to the dietary balance of tribal communities.

**Conclusions:**

Considering the importance of wild wetland plants in local sustenance, it is suggested to protect their habitats, develop domestication protocols of selected species, and build programs for the long-term management of wetland areas by involving local people. Some medicinal plants may also be used to develop into modern medicines.

## Background

Wetlands are a major feature of the landscape in all parts of the world, covering nearly 6% of its area (i.e. 8.6 million km^2^) [1]. They are the ecotones between the terrestrial and aquatic ecosystems, have unique hydrologic functions, and are extensively utilized for the supply of food, medicine, etc. along with shelter, thus forming dynamic and significant ecosystems needed by all living beings. Such lands include bog, fen, marsh, peatland, moor, swamps, bottomland or mangrove forest areas that may be wet year round or during certain periods of time. It is estimated that, globally, wetlands support goods and services worth US$70 billion per annum [2]. Unfortunately, most of the wetlands and water bodies are under increasing threats as they are drying rapidly due to various man-made impacts [3]. Many of them are now transformed into other land forms, such as paddy fields, human settlements and sites for developmental projects.

Wetlands in India, though, comprise of just < 5% of the total geographical area, they are identified as the richest and most fascinating biomes that support one-fifth of the country's total biodiversity [2]. The Indian landscape is dotted with 4290 large lakes and innumerable small water bodies [4]. These aquatic life forms play an important role in supplementing human diet and nutritional balance; besides, they also support the livelihood and income of a considerable section of society living around them. Unfortunately, there is little recognition of wetland landscapes for their current and potential value in supplying dietary food items. As 38% of the wetlands in the country have been lost in the past 10 years and many more are under threat, there is a need to take up ethnobotanical surveys of important resources that are used locally so that an action line can be developed to protect the ones in extensive use [5].

The Northeast India falls under Indo-Burma global hotspot, the area harbours large number of wetlands. Tribal communities of the region have comprehensible knowledge on use of wetland species and highly dependent on them for their livelihood, though the information on such knowledge is scanty which otherwise may form a basis for their conservation along with sustainable management. Considering that an investigation was done in wetlands of Manipur state in Northeast India with a focus to assess reliance of communities on wetland plants for subsistence and commercial needs. The aim of the study was not only to document community knowledge on wetland plants but also to draw inferences for improving livelihood of communities from these plants along with their conservation. We specifically addressed- (i) what are most common edible wetland plant species that are either used for food and/or medicinal purposes, (ii) which species traded for income generation, (iii) what are the nutritional values of edible plants, (iv) which species need immediate attention for conservation as per local perception as well as based on the extent of the pressure, and (v) what is the cultural significance of these species to tribal communities. It is expected that the study will not only documents the local knowledge for the use of these plants that may be lost in the near future as traditional cultures are eroding day by day, but also helps in maintaining a linkage between local culture and its ecosystem, which is of utmost concern for the conservation of the local environments.

## Materials and methods

### Study Area

**Figure 1:**
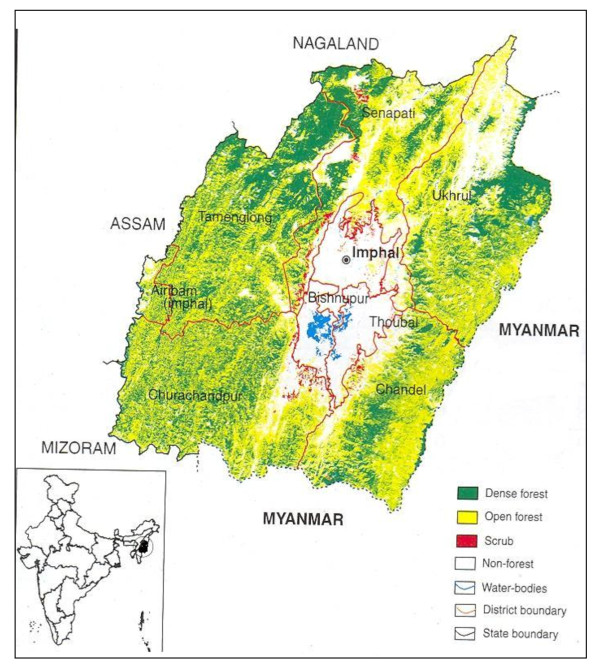
**Location map of the wetland study sites in Manipur state, Northeast India**.

Seven northeastern states of India (namely Arunachal Pradesh, Assam, Meghalaya, Manipur, Mizoram, Nagaland, and Tripura) form an integral part of the Indo-Burma centre of biodiversity hotspot of global significance [6]. The Manipur state (23°27' to 25°41' N latitude and between 93°61' to 94°48' E longitude) comprises an area of 22, 327 km^2 ^and administratively it is divided into 9 districts, of which 4 districts (viz. Imphal-East, Imphal-West, Thoubal and Bishnupur) form the lowland valleys while the rest 5 districts are upland areas (Figure 1). The hilly terrains surround a saucer-shaped centrally located valley called the Manipur/Imphal valley, where most of the wetlands are located [7] (Figure 1). The state which is rich in both cultural and biological diversity has four major ethnic communities, viz. Meitei (Hindu), Naga and Kuki (both Tribal community) and Pangal (Muslim). The Meitei is the dominant non-tribal community covering 92% of the total population in the valley area. The majority populations speak 'Manipuri' language which is included in the 8^th ^Schedule of the Indian Constitution. The main occupation of the people is agriculture, which is also associated with a high demand of natural resources and has culinary skill in day-to-day food demands. The trade of wild vegetables is considered an alternative source of income mainly done by women folks. The women also play a significant role in socio-cultural and economical fronts.

The state covers 529 km^2 ^area under wetlands (locally known as *pat*) are mainly located in lowland areas. There are 155 wetlands in the state comprising 21 lakes, 2 ox-bows, 2 reservoirs and 130 water-logged sites [8,9]. Loktak Lake is the biggest of all (286 km^2 ^area at an altitude of 769 m asl), and is also identified as one of the Ramsar sites of global significance. The wetlands are closely linked with the tribal communities for cultural, social and economic values, and are often considered a lifeline of the rural poor [10]. The tribal communities collect a large variety of edible and other useful plants from the wetlands. These wetlands also supply fishes, edible insects, plants for household consumption and medicine to the locals. The villagers also sell a large variety of edible wetland plants in the local markets to earn their livelihood.

A total of seven major wetlands from the four lowland districts (viz. Imphal-East, Imphal-West, Thoubal, and Bishnupur) were chosen for this study as they supply the bulk of edible aquatic plants in Manipur state, viz., Loktak-pat (Location 24°25'N & 93°46'E; Area 288.98 km^2^)[11], Sanapat-pat (Location 24°40'N & 93°51'E; Area 81 km^2^)[12], Uttra-pat (Location 24°25'N & 93°45'E; Area 0.37 km^2^)[13], Pumlen-pat (Location 24°20'N & 93°50'E; Area 31.88 km^2^)[14], Ikop-pat (Location 24°31'N & 93°52'E; Area 13.5 km^2^)[15], Waithou-pat (Location 24°41'N & 93°58'E; Area 0.99 km^2^)[16] and Poirou-pat (Location 24°40'N & 93°58'E; Area 0.16 km^2^)[17]. The study area recorded high annual rainfall (1900-2000 mm), and during rainy season (June-September) all wetlands received significant increase in water levels. During winter (December-February), however, the water level receded to minimum so much so that at places only 1/4^th ^of the land is covered with water. The maximum temperature of the study area ranged between 20°C to 31°C and minimum of 3.6°C to 23.5°C. High humidity recorded throughout the year which varied from 74% in March-May to over 90% during rainy season.

## Methods

### Edible wetland plants, their habit, and dietary use

The methods employed in this study were designed with the purpose of providing baseline information on the use of wetland edible plant species by tribal communities through detailed surveys during 2003 to 2006. Before undertaking the study prior informed consent was taken from the community and village heads to conduct surveys in lake and villages. After having a thorough discussion with communities seven wetlands were visited regularly on monthly basis to conduct field surveys. Purposive sampling procedure was used to study the wetland plants with relation to community dependence on them for subsistence and commercial needs. The criterion was to understand and explore best possible information on edible, medicinal, traded and cultural correlates on wetland plants from a wide range of user communities including different age groups and genders so that appropriate inference can be generalized regarding wetland plants. Detailed information was gathered using formal, informal and extensive interactions with the wetland-dwellers from both genders and with all age groups (from 20-60 years old) those involved in collection and marketing of wetland plants [18]. The questions were open ended so as to get maximum discussion from the interviewee. The inquiries comprised plants being collected from different wetlands for food and/or medicinal purposes, their local names, mode of collection, plant parts used, distribution pattern, availability periods, quantity collected, consumption/household/year, monetary value of the household consumption, management practices (if any), processing of plants before consumption, and the community demands and supply patterns of major species. The dependence of the population on edible aquatic plants and income generated from them was also estimated. A total of 224 persons were interviewed in this process.

### Marketing of edible wetland species

Three major markets of Manipur state, viz. Imphal, Bishenpur, and Thoubal were surveyed at least twice in a month for one year for analyzing marketing pattern of edible wetland species. All wetland species that brought to the market was studied for its availability period, quantity brought to the market, number of vendors involved in selling of species, trend of market availability of species, extraction pattern of species, and pressure on the resources using standard methods [19,20]. Surveys were conducted at the peak market hours between 7.00-10.00 am and 2.00-6.00 pm. At least 4-5 hours was spent in the market during every field visit. Information was gathered on the plant parts used and quantities sold, number of retailers in the market, prices and total volume available for each species, and these data were used for assessing the net quantities sold and the value of the products [21,22]. All the vegetable vendors were counted physically. For market survey a total of 96 vendors were interviewed in detail at three markets. The site of the individual vendor was almost fixed. Semi-structured questionnaires were used during the interview to collect desired data. The quantity of the plants sold was physically counted and converted into weight basis, which later on pooled on vendor basis and finally estimated on market basis. Variations in quantity and prices for each species were also gathered among three studied markets. The market price of each item was recorded on a temporal cycle and calculated the average value separately for each market. The data collected were interpreted and pooled on a whole-year basis based on which the total quantity sold and gross income from the trade was calculated [19,20,23].

### Wetland medicinal plants, their use pattern and processing

The information on species used for medicinal purposes was also gathered through questionnaires and individual interviews with resource persons and user groups (n = 120). Detailed documentation was performed for plant parts used, processing and application of the plant product. The samples of all the species were collected and identified as to their scientific names and families with the help of experts and by matching the samples with available herbaria (Botanical Survey of India) and scientific literature [24-26]. The vouchers were collected for all species and deposited in the herbarium of CSIR-NEIST (Formerly Regional Research Laboratory), Substation, Lamphelpat, Imphal, Manipur, India.

### Nutritive value of selected species

The most commonly used and marketed wetland species were estimated for their nutritional values, such as protein, fat, carbohydrate, phosphorus, potassium, sodium, iron, magnesium, copper, and zinc, using standard methods [27-29]. Samples for different species (*n *= 3) were oven dried at 60°C till a constant weight and thereafter ground separately to fine powder for laboratory analysis. Crude fat was determined by extracting a known weight of the powdered plant material with petroleum ether using Soxhlet apparatus. The carbohydrate of the samples was estimated by the Anthrone method. Acid detergent lignin was determined using Fibretech apparatus by removing the fat of a known weight of plant sample with acetone (cold extraction) and with acid detergent solution (hot extraction). The nitrogen was determined using the micro-Kjeldhal method. Phosphorus was determined through the calorimetric method while potassium and sodium through flame photometer. The micronutrients (Fe, Mn, Cu, Zn) were determined by digesting the plant samples in a tri-acid solution of HClO_4_, HNO_3_, and H_2_SO_4_, and passing it through an atomic absorption spectrophotometer using separate lamps for different micronutrients. Further details of the analysis are given [27-29].

### Species preference ranking and conservation concerns

The community perception on species use and taste, availability in natural habitats and conservation status was gathered for various edible wetland plants using random sampling method. The community observations (*n *= 76) were ranked in a scale of 1 to 4 from least to highly preferred category. Local names and selected live samples were taken during the study period for discussion. Twenty five households each for Thoubal and Bishnupur districts and 26 households for Imphal (West & East districts) were interviewed. The purpose of the data collection was explained to the interviewee and Prior Informed Consent (PIC) was taken. In the PIC, communities were assured to provide agro-technology of selected potential plant species for domestication some of which are available in the Institute (CSIR-NEIST). For assessing 'Use' and 'Taste' status the ranking was done with the community members as: 4- most preferred, 3- commonly preferred, 2- preferred but not so common, and 1- occasionally used.

For 'Availability' status the species was ranked based on field observations of the authors, market availability trend and interaction with collectors and user groups as: 4- extensively available, 3- commonly available, 2- available but not so common, 1- rare; while for 'Conservation' status, the scale ranked as 4- for the species whose conservation is highly demanded, 3- conservation urgently demanded, 2- conservation required but not so urgent, and 1- not required at present. Such ranking of the species found favour to understand community perception on the use of the species [20].

### Data analysis

Simple statistical procedure was applied for testing differences among households, villages and market respondents to wetland species data inventory and number of species used. As the data gathered for the pattern of species use and availability, collection, distribution pattern, and dietary consumption of species was qualitative, therefore the texts of interviews, group discussions, and key informants' discussions were collectively analyzed with direct field observations. For selected variables (e.g. consumption/household/year, monetary value of the household consumption, income/household/year, quantity sold in the market, nutrient content of species) having scale values, means were compared to determine levels of variation. The difference in market prices of species were presented as a range, while those of quantity sold, revenue generated and nutrient content were provided as average mean values (± SD). The information on community ranking of wetland edible plant species for their use, taste, availability and conservation status was qualitative, which was ranked in a scale of 1 to 4 to bring it to quantitative form. The data was ethnographically evaluated based on communities' perception, opinion and attitude about wetland plant resources so that the management and conservation exercises can be addressed in the area in near future.

## Results

### Edible wetland plants

**Figure 2:**
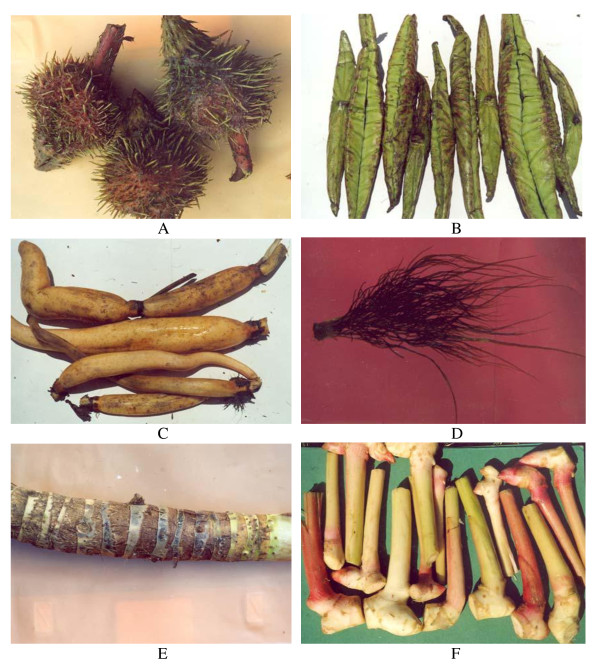
**Wetland edible plants of Manipur state, Northeast India (A. Spiny fruits of *Euryale ferox*; B. Tender rolled leaves of *Nelumbo nucifera*; C. Roots of *Nelumbo nucifera*; D. Tuff plant of *Lemaniea australis*- a red alga; E. Rhizomes of *Hedychium coronarium*, and F. Corm of *Alocasia cuculata*)**.

A total of 83 wetland plant species were recorded from seven studied wetlands of Manipur state, of which 51 plant species had economic uses to the tribal communities in the form of edibles, medicinal and other uses, and described in this investigation. These 51 edible plant species varied from 42 genera and 25 families (Table 1). All species had a common name that explains the prevalent use of these species in the local system. All edible species predominantly exhibited herbaceous life forms with different types of habits, from delicate to gregarious, creeping, prostrate, slender, and rhizomatous types (Table 1). *Lemanea australis*, an alga, is found submerged while *Jussiaea repens, Pistia stratiotes*, and *Neptunia oleracea *were free floating. The species were consumed for their shoots or aerial part (26 species), rhizomes/roots/corms (10 species), flowers/inflorescence (7 species), leaves (6 species), and fruits (2 species) (Table 1). *Euryale ferox *(21a), *Nelumbo nucifera *(Figure 2b, c), *Colocasia esculenta*, and *Oenanthe javanica *were the most commonly consumed. The use of *Lemanea australis *(Figure 2d) was selective because of its cost and low availability as the distribution was restricted to small pockets at the confluence of the rivers Chakpi and Sugnu in South Manipur. *Hedychium coronarium *(Figure 2e) was solely collected from the wild habitat while *Alocasia cuculata *(Figure 2f) was introduced to farmers individual pond.

**Table 1:** Common wetland edible plants, their habit, market prices and dietary uses from Manipur state, northeast India.

Botanical name (Family)	Local name	Plant habit	*Market price (Rs/kg)	Dietary use and preparation#	Voucher Number
*Alocasia cuculata *Schott. (Araceae)	Singju-paan	Rooted herb	Corm (15-20)	Corm cooked with fermented soybean and eaten or prepared traditional salad called as *singju *(a mixture with fermented fish, chilli and other plants).	0003 NEIST(M)
*Alpinia galanga *Willd. (Zingiberaceae)	Kanghoo	Rhizomatous herb	Rhizome (15-20)	Rhizome decoction smashed with fermented fish and chilli and eaten during both lunch and dinner.	0004 NEIST(M)
*Alpinia nigra *(Gaertn) Burtt (Zingiberaceae)	Pullei	Rhizomatous herb	Rhizome (15-20)	Rhizome boiled with potato and prepared chutney called as *eronba-*smashed with potato, fermented fish and chilli (also Used in religious ceremonies, symbolic of Manipuri New Year).	0005 NEIST(M)
*Alternanthera philoxeroides *Griseb. (Amaranthaceae)	Kabo-napi	Gregarious herb	HH	Tender shoot cooked as a traditional food item called as *ootti*-cooked many vegetables together with a pinch of soda -Na2CO3.	0006 NEIST(M)
*Amomum aromaticum *Roxb. (Zingiberaceae)	Namra	Rhizomatous herb	Rhizome (15-20)	Rhizome as a constituent in the preparation of *eronba*.	0010 NEIST(M)
*Cardamine hirsuta *Linn. (Brassicaceae)	Chantruk-maan	Delicate herb	HH	Shoot cooked-eaten occasionally.	0013 NEIST(M)
*Centella asiatica *(Linn.) Urban (Apiaceae)	Peruk	Creeping herb	Aerial part (8-10)	Whole plant is boiled, smashed with potato and fermented fish and eaten.	0014 NEIST(M)
*Colocasia esculenta *(L.) Schott (Araceae)	Paan	Herb	Corm (10-20)	Corm and leaf cooked-eaten as *ootti*.	0021 NEIST(M)
*Commelina bengalensis *Linn. (Commelinaceae)	Wangden-khoibi	Straggling herb	HH	Shoot cooked-eaten occasionally (also used as fodder).	0022 NEIST(M)
*Dryopteris marginata *(Wall.) Christ (Dryopteridaceae)	Lai-changkhrang	Straggling herb	Shoot (8 - 10)	Tender shoot fried-eaten.	0110 NEIST(M)
*Eclipta alba *(L.) Hassk. (Asteraceae)	Uchi-sumban	Prostrate herb	HH	Shoot cooked-eaten occasionally.	0029 NEIST(M)
*Eleocharis dulcis *Linn. (Cyperaceae)	Kokthum	Rooted herb	Root (20-25)	Root cooked with molasses & eaten as snacks.	0109 NEIST(M)
*Enhydra fluctuans *Lour. (Asteraceae)	Komprek-tujombi	Herb	HH	Shoot cooked-eaten or raw as *singju*.	0033NEIST(M)
*Euryale ferox *Salisb. (Nymphaeaceae)	Thangjing	Rooted herb	Fruit (20-25)	Fruit cooked-eaten or raw as *eronba*; leaf petiole is eaten as salad.	0034 NEIST(M)
*Fagopyrum esculentum *Moench. (Polygonaceae)	Wakha-yendem	Herb	Shoot (5-8)	Leaf and shoot cooked-eaten as vegetables (also used as fodder for goats).	0035 NEIST(M)
*Gynura cusimbua *(D. Don) Moore (Asteraceae)	Tera-paibi	Herb	HH	Shoot cooked-eaten occasionally.	0111 NEIST(M)
*Hedychium coronarium *Koenig. (Zingiberaceae)	Lok-lei	Tall herb	Rhizome (25-30)	Rhizome cooked and prepared *eronba*.	0036 NEIST(M)
*Hedyotis auricularia *Linn. (Rubiaceae)	Langban-koukha	Creeping Herb	HH	Tender shoot cooked as *ootti*.	0039 NEIST(M)
*Ipomoea aquatica *Forsk. (Convolvulaceae)	Kolamni	Herb	Shoot (5-6)	Shoot cooked-eaten.	0044 NEIST(M)
*Jussiaea repens *Linn. (Onagraceae)	Ishing-kundo	Floating herb	HH	Shoot cooked as *ootti*.	0112 NEIST(M)
*Jussiaea suffruticosa *Linn. (Onagraceae)	Tebo	Straggling Herb	HH	Shoot cooked as *ootti*.	0113 NEIST(M)
*Lemanea australis *Atkins. (Rhodophyceae)	Nung-sam	Submerged minute herb	Whole plant (1200-1500)	Dried and roasted filaments prepared chutney (the plant produce characteristic fishy smell when roasted in fire- used as fish substitute).	0047 NEIST(M)
*Marsilea minuta *Linn. (Marsileaceae)	Ishing-yensang	Slender herb	HH	Aerial plant parts cooked as *ootti*.	0051 NEIST(M)
*Narenga porphyrocroma *Bor. (Poaceae)	Singhut-kambong	Under shrub	HH	Infected (with fungus) inflorescence is roasted in the fire, smashed with molasses and rice and eaten.	0114 NEIST(M)
*Nasturtium indicum *Linn. (Brassicaceae)	Uchi-hangam	Delicate herb	HH	Cooked-eaten as vegetables.	0015NEIST(M)
*Nelumbo nucifera *Gaertn. (Nymphaeaceae)	Thambal	Rooted-hydrophyte	Fruit, leaf, flower (12-16), Root (15-20)	Flower, tender shoot, leaf and roots eaten raw as salad; root cooked with molasses & eaten as snacks.	0053 NEIST(M)
*Nephalium indicum *Linn. (Asteraceae)	Phunil	Slender hispid herb	Shoot (5-10)	Shoot is prepared *eronba*.	0116 NEIST(M)
*Neptunia oleracea *Lour. (Mimosaceae)	Ikaithabi	Floating	Shoot (25-35)	Shoot cooked as *eronba *or eaten raw as *singju*.	0054 NEIST(M)
*Nymphaea alba *Linn. (Nymphaeaceae)	Tharo-angouba	Rooted Hydrophyte	Flower: 20 Fruit, petiole: 10	Flower and petiole eaten as salad or *singju *(also used as religious offering).	0117 NEIST(M)
*N. nouchali *Burma f. (Nymphaeaceae)	Tharo-angangba	Rooted Hydrophyte	Flower (15-20) Fruit, petiole (10)	Flower and petiole eaten as salad or *singju *(also as religious offering).	0055 NEIST(M)
*Nymphaea pubescens *Willd. (Nymphaeaceae)	Tharo-ashangba	Rooted Hydrophyte	Flower (18-20) Fruit, petiole (10)	Flower and petiole eaten as salad or *singju *(also as religious offering).	0118NEIST(M)
*Nymphaea stellata *Willd. (Nymphaeaceae)	'Thariktha'	Rooted Hydrophyte	Flower (20) Fruit, petiole (10)	Flower and petiole eaten as salad or *singju*.	0056NEIST(M)
*Nymphoides indicum *(L.) Kuntze (Gentianaceae)	Thariktha-macha	Rooted slender hydrophyte	Petiole (10-14)	Leaf petiole eaten as *singju *or as *eronba*.	0057 NEIST(M)
*Oenanthe javanica *(Blume) DC (Apiaceae)	Komprek	Swampy slender herb	Shoot (15-20)	Shoot & leaf is one of the best and preferred species used in the preparation of *singju*.	0060 NEIST(M)
*Oxalis corniculata *Linn. (Oxalidaceae)	Yensil	Delicate herb	HH	Plant cooked with seeds of pea (*Pisum sativum*) and eaten during both lunch and dinner.	0062 NEIST(M)
*Persicaria posumba *(Buch- Ham ex D. Don) H. Gross. (Polygonaceae)	Kengoi	Delicate herb	Aerial part (15- 20)	Plant cooked with dry fishes and eaten.	0119 NEIST(M)
*Pistia stratiotes *Linn. (Araceae)	Kang-jao	Floating herb	HH	Leaf cooked as *ootti *or as *eronba *(also used as fodder and feed to poultry).	0120 NEIST(M)
*Plantago erosa *Wall. (Plantaginaceae)	Yempat	Herb	HH	Leaf cooked-eaten occasionally.	0068 NEIST(M)
*Polygonum barbatum *Linn. (Polygonaceae)	Yelang	Silvery coloured herb.	Shoot (15-20)	Shoot cooked-eaten or raw as *singju*.	0069 NEIST(M)
*Polygonum chinense *Linn. (Polygonaceae)	Angom-yensil	Slender herb	HH	Shoot cooked along with peas.	0121 NEIST(M)
*Polygonum molle *D. Don. (Polygonaceae)	Leibung-tharam	Tall herb	Shoot (6-7)	Leaf and shoot cooked-eaten (also used as fodder plant).	0071 NEIST(M)
*Polygonum plebejum *R. Br. (Polygonaceae)	Okthum	Slender herb	HH	Shoot cooked-eaten occasionally.	0122 NEIST(M)
*Rumex nepalensis *Spreng (Polygonaceae)	Torong-khongchak	Herb	HH	Leaf and shoot cooked eaten (also used as poultry feed and animal fodder).	0083 NEIST(M)
*Sagittaria sagittifolia *Linn. (Alismataceae)	Koukha	Slender erect Herb	Root (22-28)	Root cooked-eaten along with molasses, and also prepared *eronba *and traditional *pokada *(fried in oil).	0086 NEIST(M)
*Schoenoplectus lacustris *(L.) Palla (Cyperaceae)	Kouna	Tall spongy Herb	(20-30 per 100 tillers)	Tender shoot eaten raw occasionally by children with slightly bitter taste (also used in handicrafts & mats used in religious ceremonies and household needs).	0087 NEIST(M)
*Spilanthes acmella *Hook. f. (Asteraceae)	Chin-lengbi	Straggling herb	Shoot (5-8)	Shoot cooked-eaten.	0090 NEIST(M)
*Stellaria media *(L.) Vill. (Caryophyllaceae)	Yerum-keirum	Delicate herb	Shoot (15-20)	Shoot cooked-eaten as vegetable.	0091 NEIST(M)
*Trapa natans *Linn. (Trapaceae)	Heikak	Rooted hydrophyte	Fruit (8-13)	Fruits cooked-eaten or as raw; petiole eaten as *eronba *and *singju*.	0094 NEIST(M)
*Viola pilosa *Blume (Violaceae)	Huikhong	Small herb	Shoot (12-18)	Shoot cooked with dried fish and eaten.	0095 NEIST(M)
*Zizania latifolia *Turcz. *ex* Stapf. (Poaceae)	Ishing-kambong	Erect tall Herb	Inflorescence (28-35)	Infected inflorescence roasted in fire and eaten along with molasses and rice (shoot of this plant is best fodder for brow-antlered deer - *Cervus eldi eldi - *a critically engendered species).	0099 NEIST(M)

* HH = consumed at household level only and not traded in markets

# text in parenthesis in 'Dietary use and preparation' column shows other utility of the species

The communities used different modes to consume these species (Table 1). Fresh plucking of the vegetables just before cooking was the most preferred mode to use the species and the people have traditional culinary skills for the preparation of traditional delicacies, such as *ootti, eronba, singju*, etc. *Singju*, the most common traditional food dishes was made by mixing wetland edible species with fermented fish, chilli, and other plants; *eronba *was prepared by boiling plant parts and smashing it with potatoes, chilli, and fermented fish before consuming; while *ootti *was prepared by boiling vegetables with a pinch of sodium bicarbonate before eating (Table 1). At least one item in an everyday meal was an essential constituent of the local diets, which also explained the importance of wetland plants in the local system.

### Trade of wetland species

The market survey data on the number of vendors selling wetland edible plants revealed a total of 1500 vendors were registered to sell their produce in the markets. It was recorded that nearly 502 vendors were selling wild edible plants in Imphal, 134 in Bishenpur and only 10 vendors in Thoubal markets. Of the total 51 edible plant species recorded in this investigation, 27 species were traded in the three studied markets, while 24 species consumed at household level only (Table 2). Women were the major stakeholders in the trade; they collect different species from the wetland areas and sold them directly in the local markets. The selling prices varied with species, with season and market to market.

**Table 2:** Most common wetlands edible plants traded in three markets of Manipur state.

Plant species	Plant parts used	Imphal market		Bishenpur market		Thoubal market	
		Quantity sold (ton/annum)	Total revenue (Rs.)	Quantity sold (ton/annum)	Total revenue (Rs.)	Quantity sold (ton/annum)	Total revenue (Rs.)
*Alocasia cuculata*	Corm	2.88	51842	0.60	4800	0.06	1225
*Alpinia galanga*	Rhizome	0.13	4460	-	-	-	-
*Alpinia nigra*	Rhizome	2.16	32390	-	-	-	-
*Amomum aromaticum*	Rhizome	1.44	46060	0.32	4460	0.19	7690
*Amomum *sp.	Rhizome	1.44	28812	0.06	345	0.09	1960
*Cardamine hirsuta*	Shoot	-	-	0.10	685	-	-
*Centella asiatica*	Wh. plant	1.73	15530	0.80	12790	0.16	1910
*Colocasia esculenta*	Corms	7.56	113385	4.03	40330	6.91	69140
*Eleocharis dulcis*	Root	0.86	19012	0.04	345	0.48	12005
*Euryale ferox*	Fruits	31.10	248820	4.61	55270	7.68	76780
*Fagopyrum esculentum*	Shoot	0.10	590	-	-	-	-
*Hedychium coronarium*	Rhizome	2.02	56445	0.48	3575	0.96	2400
*Ipomoea aquatica*	Shoot	1.51	11320	0.67	3380	0.67	6715
*Lemanea australis*	Plant	0.007	9700	-	-	0.002	3235
*Nephalium indicum*	Shoot	0.03	295	-	-	-	-
*Nelumbo nucifera*	Fruits, Roots	4.32	77765	0.79	8330	0.36	11515
*Neptunia oleracea*	Shoot	0.77	23030	0.58	14405	0.10	2597
*Nymphaea alba*	Petiole, flower	0.96	17300	1.80	9015	0.20	784
*Oenanthe javanica*	Shoot	3.84	46060	2.69	26900	1.92	51840
*Persicaria posumba*	Wh. plant	0.96	19210	-	-	-	-
*Polygonum barbatum*	Shoot	1.44	21610	0.32	7985	2.16	47530
*Polygonum molle*	Shoot	0.08	490	-	-	-	-
*Sagittaria sagittifolia*	Roots	1.28	28175	1.34	30920	0.96	1910
*Stellaria media*	Shoot	0.03	540	-	-	0.30	4510
*Trapa natans*	Fruits, Leaf	0.90	9015	0.45	4460	0.28	3380
*Viola pilosa*	Wh. plant	0.36	4312	0.02	590	-	-
*Zizania latifolia*	Infected culms	0.72	21610	0.48	12495	0.14	4310

		**68.627**	**907, 778**	**20.18**	**241, 080**	**23.622**	**311, 436**

1US$ = Rs. 45.00 (at the time of survey), Wh. plant = Whole plant

In 2005-2006, Imphal market received a total volume of 68.63 tons of edible wetland plants involving a business of over Rs. 9, 07, 778 (Rs. 45 = 1US$). Thoubal received 23.62 tons of vegetables involving Rs. 3, 11, 436 while Bishenpur received 20.18 tons with a trade of Rs. 2, 41, 080 (Table 2). Thus, in terms of total volume of edible plants received, Imphal (the state capital) market recorded at least 2.85 times higher than Thoubal and 3.4 times higher than Bishenpur market. Nearly 70% of the annual income from the wild edible plants of the three markets was generated by seven species (*Euryale ferox, Colocasia esculenta, Oenanthe javanica, Nelumbo nucifera, Polygonum barbatum, Hedychium coronarium*, and *Sagittaria sagittifolia)*, while the rest of the species contributed just 30% (Table 2). Among individual species, *Euryale ferox *was sold in the highest quantity (43.39 tons), followed by *Colocasia esculenta, Oenanthe javanica*, and *Nelumbo nucifera. Lemanea australis *was the most expensive species, as recorded in Imphal and Thoubal markets; contrarily, *Fagopyrum esculentum *and *Polygonum molle *were recorded as the cheapest among all the vegetables sold (Table 2).

### Medicinal wetland plants

Of the total of 51 wetland edible plant species recorded, 38 species also used for medicinal purpose in traditional systems (Table 3). It was interesting to note that these species were used to cure some 22 diseases and ailments. Among the most commonly used plant parts for medicinal purposes, the use of the whole plant/shoots was most common (15 species), which was closely followed by the use of leaves (14 species) (Table 3). The other plant parts used were petiole (2 species), flower/inflorescence (2 species), fruit/seed (4 species), and root/rhizome (3 species). The most common practice for the use of the plants for medicinal purposes was to make paste, decoction or powder, or to boil or eat raw (Table 3). An investigation with the local people revealed that the most common diseases that were treated with local medicinal plants were cuts and injuries (9 species); boils, burns and wounds (9 species); cough and fever (6 species); indigestion, dysentery and intestinal infections (6 species); diabetes (5 species); blood pressure and circulation problems (3 species); earache and insect bites (2 species); and muscular sprains, intestinal worms, leucoderma, jaundice, and stomach ulcers (1 species each). Considering the cheapest and most effective means, all these species were highly popular in the local systems (Table 3).

**Table 3:** Edible plants with medicinal utility from wetlands of Manipur state, India.

Scientific name	Local name	Parts eaten	Other traditional uses
*Alpinia galanga*	Kanghoo	Rhizome	Paste is eaten to treat intestinal worms; abortifacient and applied in leucoderma.
*Alternanthera philoxeroides*	Kabo-napi	Leaf	Paste is applied on cut and injuries.
*Amomum aromaticum*	Namra	Seed	Powder is taken to control high blood pressure.
*Cardamine hirsuta*	Chantruk-man	Leaf	Paste is applied on cut and injuries.
*Centella asiatica*	Peruk	Whole plant	Extract is tonic, given in cough and diabetes.
*Colocasia esculenta*	Paan	Petiole	Juice is applied on cut and injuries.
*Commelina bengalensis*	Wangden-khoibi	Whole plant	Decoction paste is applied on boils and burns. Hot fermented plant is applied on muscular sprain.
*Dryopteris marginata*	Lai-changkhrang	Leaf	Paste used for cuts, injuries and on boils/burns.
*Eclipta alba*	Uchi-sumban	Plant	Plant is astringent aphrodisiac and expectorant.
*Enhydra fluctuans*	Komprek-tujombi	Fresh plant	Extract is given in diabetes.
*Euryale ferox*	Thanging	Fruit, leaf	Raw fruit eaten against diabetes; leaf petiole paste applied on burns and boils.
*Gynura cusimbua*	Tera-paibi	Leaf	Paste applied on injuries, cooked leaf eaten to cure diabetes and high blood pressure.
*Hedychium coronarium*	Lok-lei	Rhizome, leaf	Paste of rhizome is eaten against cough, fever; leaf extract is given against throat complaint.
*Hedyotis auricularia*	Langban-koukha	Leaf	Extract is given against dysentery and cough.
*Ipomoea aquatica*	Kolamni	Shoot	Boiled leaf extract is used as ear-drop to treat ear-ache; leaf paste is applied on insect bite.
*Jussiaea repens*	Ishing-kundo	Leaf	Paste is applied on cut and injuries and also on aching gums.
*Jussiaea suffruticosa*	Tebo	Leaf	Paste is applied on fresh cut and injuries.
*Lemanea australis*	Nung-sam	Whole plant	Plant is boiled and the soup is taken to cure diabetes.
*Marsilea minuta*	Ishing-yensang	Shoot/leaf	Plant paste is applied on boils and burns.
*Nasturtium indicum*	Uchi-hangam	Leaf	Paste is applied n cuts and skin diseases.
*Nelumbo nucifera*	Thambal	petiole	Paste of petiole is applied on boils and burns.
*Neptunia oleracea*	Ikaithabi	Shoot	Eaten raw in dysentery and intestinal infections.
*Nymphoides indica*	Thariktha-macha	Leaf	Extract is applied on boils and burns.
*Oenanthe javanica*	Komprek	Shoot	Boiled in little water and the filtrate is used as ear-drop to cure ear-ache.
*Oxalis corniculata*	Yensil	Plant	Paste applied on boils and burns, cooked eaten in dyspepsia.
*Persicaria posumba*	Kengoi	Plant	Eaten to cure diabetes, piles and intestinal disorder.
*Pistia stratiotes*	Kang-jao	Leaf	Paste is applied on boils and blisters.
*Plantago erosa*	Yempat	Seed, plant	Powder with little honey is given in fever; Boiled plant is used in muscular sprain.
*Polygonum barbatum*	Yelang	Fresh shoot	Paste is taken to treat stomach disorder and dysentery.
*Polygonum chinense*	Angom-yensil	Plant	Paste is given in cut and injuries, fever and dyspepsia.
*Polygonum molle*	Leibung-tharam	Shoot	Crushed shoot and applied on wounds.
*Polygonum plebejum*.	Okthum	Plant	Paste is applied on injuries.
*Sagittaria sagittifolia*	Koukha	Root	Paste along with honey is given in cough.
*Spilanthes acmella*	Chin-lengbi	Flower	Paste is given to treat jaundice and sore throat.
*Stellaria media*	Yerum-keirum	Fresh shoot	Plant decoction is applied on fresh wounds, skin itching and nose bleeding.
*Trapa natans*	Heikak	Fruit	Eaten for better blood circulation.
*Viola pilosa*	Hui-khong	Leaf	Cooked and eaten to cure cough, running nose and stomach ulcer.
*Zizania latifolia*	Ishing-kambong	Inflorescence	Infected inflorescence is roasted in fire and eaten to treat indigestion.

### Nutrient status of edible wetland plants

A total of 21 wetland edible plant species were analyzed for twelve different nutritional parameters (Table 4 and 5). The lignin content was estimated high in *Viola pilosa, Hedyotis auricularia, Oxalis corniculata*, and *Lemanea australis *(Table 4). For other species, the lignin content varied from 1 to 18%. The fat content of all investigated species varied from 0.1% to 10%, being maximum in *Lemanea australis *and minimum in *Nelumbo nucifera*. The carbohydrate content ranged between 3.4 and 32.5% among different species; it was recorded high in *Lemanea australis, Nelumbo nucifera*, and *Colocasia esculenta*. Higher protein content was recorded in young shoots of *Lemanea australis *(20.2%) and *Rumex nepalensis *(14.9%), on the contrary, it was low (2.6-2.61%) in *Alpinia galanga *and *Sagittaria sagittifolia*. The total phosphorus content was high in *Fagopyrum esculentum *and low in *Colocasia esculenta *(Table 4). The total nitrogen was estimated high in *Lemanea australis, Rumex nepalensis *and *Jussiaea repens*. The potassium content was maximum in *Zizania latifolia *(0.46%) and a minimum in *Fagopyrum esculentum *(0.016%). The sodium content was high in *Fagopyrum esculentum*, while it was low in *Eleocharis dulcis *(Table 4). All other species had intermediate range of nutrients. A total of 13 wetland species were also estimated for micronutrients (Table 5). The maximum and minimum content was recorded for iron in *Sagittaria sagittifolia *and *Polygonum barbatum*; for magnesium in *Viola pilosa *and in *Eleocharis dulcis*; for copper in *Lemanea australis *and *Alpinia galanga*; and for zinc in *Lemanea australis *and in *Marsilea minuta*, respectively (Table 5).

**Table 4:** Nutrient content of some most preferred wild edible plants from the wetlands of Manipur (values are ± SD).

Botanical name	Parts	Lignin (%)	Fat (%)	Carbo-hydrate (%)	Protein (%)	Total Phosphorus (%)	Total Nitrogen (%)	Potassium (%)	Sodium (%)
*Alpinia galanga*	Rhizome	18 ± 0.5	1 ± 0.2	4.4 ± 1.1	2.6 ± 1.11	0.58 ± 0.02	0.40 ± 0.01	0.33 ± 0.01	0.02 ± 0.001
*Cardamine hirsuta*	Shoot	6 ± 0.6	3 ± 0.6	8 ± 1.56	14.4 ± 1.22	0.66 ± 0.01	0.66 ± 0.01	0.44 ± 0.003	0.016 ± 0.00
*Centella asiatica*	Whole plant	1 ± 0.05	1 ± 0.5	7 ± 2.12	8.25 ± 0.12	0.62 ± 0.01	0.62 ± 0.008	0.33 ± 0.019	0.08 ± 0.001
*Colocasia esculenta*	Corm	9 ± 0.8	2 ± 0.3	18.5 ± 3.4	4.07 ± 1.22	0.385 ± 0.04	0.65 ± 0.001	0.34 ± 0.02	0.014 ± 0.001
*Commelina bengalensis*	Shoot	5 ± 0.6	1 ± 0.5	5 ± 1.12	9.4 ± 0.11	0.813 ± 0.11	1.5 ± 0.03	0.34 ± 0.01	0.02 ± 0.001
*Eleocharis dulcis*	Root	4 ± .02	1 ± .03	10.2 ± 2.3	6.56 ± 1.23	0.684 ± 0.040	1.50 ± 0.030	0.187 ± 0.003	0.008 ± 0.0
*Fagopyrum esculentum*	Shoot	9 ± 0.5	1 ± 0.3	4 ± 2.3	8.31 ± 0.22	0.991 ± 0.123	1.33 ± 0.01	0.016 ± 0.002	0.24 ± 0.000
*Hedychium coronarium*	Rhizome	9 ± 1	2 ± 0.5	10 ± 3.5	4.63 ± 1.11	0.92 ± 0.03	0.92 ± 0.028	0.056 ± 0.003	0.018 ± 0.003
*Hedyotis auricularia*	Shoot	70 ± 3.5	3 ± 1	7.3 ± 2.3	7.88 ± 0.22	0.4 ± 0.02	0.4 ± 0.024	0.121 ± 0.001	0.014 ± 0.001
*Ipomoea aquatica*	Shoot	10 ± 1.2	3.6 ± 0.4	4.4 ± 1.1	14.5 ± 1.22	0.6 ± 0.03	0.601 ± 0.03	0.41 ± 0.007	0.15 ± 0.013
*Jusiaea repens*	Shoot	5 ± 1.1	1 ± 0.005	11.3 ± 2.7	13.5 ± 0.23	0.96 ± 0.18	2.16 ± 0.001	0.26 ± 0.002	0.017 ± 0.000
*Jusiaea suffruticosa*	Shoot	9 ± 2.1	2 ± 0.08	6.8 ± 3	9.76 ± 0.13	0.98 ± 0.01	1.56 ± 0.006	0.134 ± 0.002	0.019 ± 0.002
*Lemanea australis*	Whole plant	23 ± 2.2	10 ± 1.4	32.5 ± 5.5	20.2 ± 0.11	-	3.18 ± 0.12	0.4 ± 0.110	-
*Marsilea minuta*	Shoot	9 ± 1	4 ± 0.8	4.8 ± 1.9	8.0 ± 1.24	0.94 ± 0.001	1.28 ± 0.021	0.373 ± 0.005	0.017 ± 0.00
*Nelumbo nucifera*	Root	-	0.1 ± .001	26.5 ± 4.3	2.78 ± 0.01	0.44 ± 0.04	0.39 ± 0.02	-	-
*Oxalis corniculata*	Whole plant	45 ± 4.7	2 ± 0.5	11.8 ± 3.4	9.2 ± 0.11	0.92 ± 0.23	1.47 ± 0.001	0.3 ± 0.007	0.018 ± 0.001
*Polygonum barbatum*	Shoot	10 ± 2.1	2 ± 0.3	3.7 ± 1.4	7.5 ± 0.11	0.44 ± 0.003	1.20 ± 0.03	0.015 ± 0.012	0.035 ± 0.005
*Rumex nepalensis*	Shoot	4 ± 0.6	2 ± 0.1	10.5 ± 2.5	14.9 ± 0.11	0.88 ± 0.04	2.38 ± 0.01	0.415 ± 0.01	0.1 ± 0.001
*Sagittaria sagittifolia*	Root	6 ± 0.7	1 ± 0.006	3.4 ± 1.3	2.61 ± 0.12	0.8 ± 0.001	0.42 ± 0.001	0.018 ± 0.003	0.12 ± 0.01
*Viola pilosa*	Shoot	76 ± 5.1	3 ± 0.9	7.5 ± 1.4	4.9 ± 0.21	0.43 ± 0.004	0.78 ± 0.002	0.35 ± 0.005	0.017 ± 0.001
*Zizania latifolia*	Inflorescence	7 ± 1.2	1 ± 0.5	13.8 ± 2.5	8.13 ± 1.23	0.78 ± 0.11	1.3 ± 0.005	0.46 ± 0.002	0.022 ± 0.005

-not analyzed

**Table 5:** Micro-nutrients in selected wetland edible plant species (values are ± SD).

Botanical name	Iron (ppm)	Magnesium (ppm)	Copper (ppm)	Zinc (ppm)
*Alpinia galanga*	1.25 ± 0.30	3.10 ± 0.67	0.03 ± 0.001	0.45 ± 0.12
*Centella asiatica*	0.85 ± 0.07	0.72 ± 0.14	0.12 ± 0.01	1.24 ± 0.23
*Colocasia esculenta*	0.58 ± 0.12	3.14 ± 0.32	0.05 ± 0.01	2.87 ± 0.25
*Jusiaea repens*	0.90 ± 0.15	2.77 ± 0.23	0.16 ± 0.01	1.13 ± 0.30
*Eleocharis dulcis*	0.67 ± 0.02	0.42 ± 0.09	0.08 ± 0.003	1.32 ± 0.13
*Jusiaea suffruticosa*	0.75 ± 0.18	0.82 ± 0.11	0.09 ± 0.02	0.33 ± 0.11
*Lemanea australis*	0.65 ± 0.22	-	31.20 ± 2.56	62.40 ± 3.50
*Marsilea minuta*	0.68 ± 0.34	0.71 ± 0.15	0.09 ± 0.02	0.21 ± 0.10
*Oxalis corniculata*	0.90 ± 0.12	1.85 ± 0.16	0.15 ± 0.10	1.13 ± 0.45
*Polygonum barbatum*	0.071 ± 0.001	2.02 ± 0.40	0.04 ± 0.01	2.95 ± 0.45
*Sagittaria sagittifolia*	1.30 ± 0.20	0.78 ± 0.21	0.14 ± 0.02	0.74 ± 0.22
*Viola pilosa*	0.66 ± 0.15	3.35 ± 0.33	0.04 ± 0.01	1.260.21
*Zizania latifolia*	0.85 ± 0.21	3.34 ± 0.21	0.12 ± 0.06	4.71 ± 1.45

- not analyzed

### Species preference ranking and conservations status

**Figure 3:**
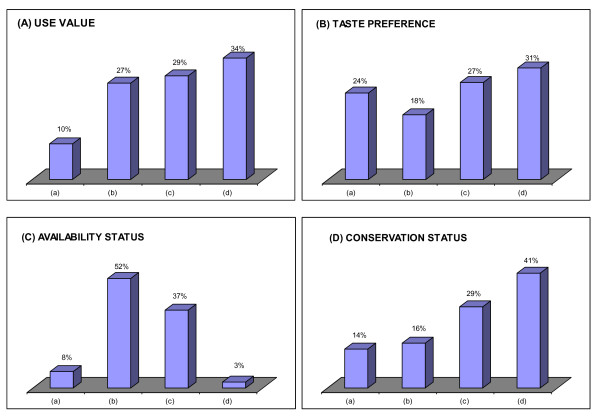
**Bar diagram showing percent Use value (A), Taste preference (B), Availability status (C) and Conservation status (D) of wetland plants in Manipur state, Northeast India (ranking for 'Use' and 'Taste' status comprised as: (a) occasionally used, (b) preferred but not so common, (c) commonly preferred, and (d) most preferred; for 'Availability' status: (a)- rare, (b) available but not so common, (c) commonly available, and (d) extensively available; and for 'Conservation' status: (a) no conservation required at present, (b) conservation required but not so urgent, (c) conservation urgently required, and (d) conservation highly required)**.

Community matrix ranking of use status, taste preference, availability status and conservation of the 51 wetland edible species is presented in Table 6 and Figure 3A to 3D. As far as use of species was concerned five species most-preferred while 14 another commonly-preferred (Table 6 Figure 3A). The most commonly used species were *Alocasia cuculata, Euryale ferox, Lemanea australis, Neptunia oleracea *and *Oenanthe javanica*. It was recorded that due to limited resource available and high collection of *Lemanea australis *in recent times, the habitat of this plant was at risk. *Lemanea australis *showed rare occurrence (Table 6). Taste wise 12 species were highly preferred while another 9 species were commonly preferred (Figure 3B). Availability status of species showed just 4 species as extensive available and another 21 species as commonly available (Figure 3C). The communities sensed that conservation of *Alocasia cuculata, Euryale ferox, Lemanea australis, Nelumbo nucifera, Neptunia oleracea, Schoenoplectus lacustris *and *Zizania latifolia *was highly demanding due to its wide use, heavy collection and market demand (Figure 3D). Considering the four ranking parameters, the most desired species were *Alocasia cuculata, Euryale ferox, Nelumbo nucifera, Neptunia oleracea *and *Zizania latifolia *(Table 6). It was interesting to note that farmers have started cultivation of *Euryale ferox, Neptunia oleracea, Alocasia cuculata, Nelumbo nucifera, Alpinia galanga *and *Colocasia esculenta *at household level in ponds while all other species were collected from wild wetland areas.

**Table 6:** Community matrix ranking for wetland edible plant species for use, taste, availability and conservation status in Manipur state, northeast India.

Botanical names	Local name	*Use status	Taste preference*	Availability status*	Conservation status*	Total score
*Alocasia cuculata*	Singju-paan	4	4	2	4	14
*Alpinia galanga*	Kanghoo	2	3	2	2	9
*Alpinia nigra*	Pullei	2	3	2	3	10
*Alternanthera philoxeroides*	Kabo-napi	1	1	4	1	7
*Amommum *sp.	Sarei	3	3	2	3	11
*Amomum aromaticum*	Namra	3	3	2	3	11
*Cardamine hirsuta*	Chantruk-maan	1	1	3	1	6
*Centella asiatica*	Peruk	3	4	3	2	12
*Colocasia esculenta*	Paan	2	2	3	2	9
*Commelina bengalensis*	Wangden-khoibi	1	1	3	1	6
*Dryopteris marginata*	Lai-changkhrang	2	2	3	1	8
*Eclipta alba*	Uchi-sumban	1	1	2	1	5
*Eleocharis dulcis*	Kokthum	3	4	2	2	11
*Enhydra fluctuans*	Komprek-tujombi	3	3	2	3	11
*Euryale ferox*	Thangjing	4	4	2	4	14
*Fagopyrum esculentum*	Wakha-yendem	3	2	3	2	10
*Gynura cusimbua*	Tera-paibi	3	1	3	1	8
*Hedychium coronarium*	Lok-lei	3	4	2	2	11
*Hedyotis auricularia*	Langban-koukha	1	1	3	1	6
*Ipomoea aquatica*	Kolamni	3	3	3	2	11
*Jusiaea repens*	Ishing-kundo	1	1	3	1	6
*Jusiaea suffruticosa*	Tebo	1	1	3	1	6
*Lemanea australis*	Nung-sam	4	4	1	4	13
*Marsilea minuta*	Ishing-yensang	1	1	3	1	6
*Narenga porphyrocroma*	Singhut-kambong	1	1	2	2	6
*Nasturtium indicum*	Uchi-hangam	1	1	3	1	6
*Nelumbo nucifera*	Thambal	3	4	3	4	14
*Nephalium indicum*	Phunil	2	2	2	1	7
*Neptunia oleracea*	Ikaithabi	4	4	2	4	14
*Nymphaea alba*	Tharo-angouba	2	2	2	2	8
*Nymphaea nouchali*	Tharo-angangba	2	2	2	2	8
*Nymphaea pubescens*	Tharo-ashangba	2	2	2	2	8
*Nymphaea stellata*	Thariktha	2	2	2	2	8
*Nymphoides indica*	Thariktha-macha	2	2	2	2	8
*Oenanthe javanica*	Komprek	4	4	2	3	13
*Oxalis corniculata*	Yensil	1	2	4	1	8
*Persicaria posumba*	Kengoi	2	3	2	2	9
*Pistia stratiotes*	Kang-jao	1	1	4	1	7
*Plantago erosa*	Yempat	1	1	3	1	6
*Polygonum barbatum*	Yelang	3	3	2	3	11
*Polygonum chinense*	Angom-yensil	2	2	3	1	8
*Polygonum molle*	Leibung-tharam	1	2	3	1	7
*Polygonum plebejum*	Okthum	1	1	3	1	6
*Rumex nepalensis*	Torong-khongchak	1	1	4	1	7
*Sagittaria sagittifolia*	Koukha	3	4	2	3	12
*Schoenoplectus lacustris	Kouna	1	1	2	4	8
*Spilanthes acmella*	Chin-lengbi	2	2	3	1	8
*Stellaria media*	Yerum-keirum	2	2	3	1	8
*Trapa natans*	Heikak	3	4	2	3	12
*Viola pilosa*	Huikhong	2	3	2	2	9
*Zizania latifolia*	Ishing-kambong	3	4	3	4	14

* The status of species in each category is rank in a scale of 4 to1 (Use and Taste status: 4-most preferred, 3-commonly preferred, 2-preferred but not so common, and 1-occassionally used; Availability status: 4-extensively available, 3-commonly available, 2-available but not so common, and 1-rare; Conservation status: 4-widely required, 3-normally required, 2-required but not so common, 1-not required at present).

## Discussion

The northeast region of India, which forms a major part of the Indo-Burma hotspot, supported considerable biodiversity, a significant share contributed by wetland areas [4,5,19]. The region represented plains, valleys and hilly terrains and the state of Manipur formed a true representative of this region, which was undertaken for detailed investigation. The wetland areas supply a wide variety of edible plants to local people for food and medicinal purposes. Besides, they also contribute other services, such as aesthetic, income, food to animals and handicraft [30,31], thus formed an area of high socio-cultural significance [10]. The people of the state have traditionally been dependent on the wild plant resources for various cultural and religious purposes for centuries [32]. A large variety of such edible plants were also sold in the markets as a means of livelihood for the rural population. The customary food habit was simple, rice being a staple item with green leafy vegetables and salad. Use of one or more wetland edible item was a compulsory part of a local meal. Although fish provided a good protein source, not many people can afford it regularly and their nutrition solely depends on the fresh green vegetables they eat. The traditional dishes (i.e. *ootti, eronba, singju*, etc.) still form an important ingredient of the local menu. Such use of edible wild plants in traditional delicacies was common among the tribal communities in the Himalayan Mountains [18]. Over 90% of species were still collected from wild habitats. The income from *Nelumbo nucifera *was high as it has multiple uses, including having a sacred value in religious ceremonies; however, the species' existence is under threat due to the conversion of its growing areas into paddy fields [30].

The total annual volume of the edible wetland plants in three markets, estimated to the tune of 113 ton/annum with a net revenue of over Rs. 9, 00, 000/-, was considerable. This volume was in addition to the quantity collected for household consumption. A large section of rural population was involved in the trade, which mainly comprised womenfolk. The most demanding species comprised *Neptunia oleracea, Lemanea australis, Sagittaria sagittifolia*, and *Zizania latifolia*, despite their limited quantities, which could be attributed to their narrow range of distribution and availability during the growing season only. Around 70% of the annual income of wetland plants in three markets was generated from seven species (*Euryale ferox, Colocasia esculenta, Oenanthe javanica, Nelumbo nucifera, Polygonum barbatum, Hedychium coronarium*, and *Sagittaria sagittifolia) *while remaining 30% income by others. As revealed by some of the vendors the availability of wetland edible plants was registering a decline in recent times, which can be attributed to destruction and shrinkage of wetland habitats at places and also due to erratic rainfall that may cause flood and/or dry condition. A possible climate change over the years also reported having an impact on agriculture, water bodies and forest areas in North east region [33].

Consumption of wild food items formed a major source of vitamins and micronutrients for people in remote rural settlements where vegetable cultivation was not much practiced [34]. The young shoots and leaves found to have high protein content. *Lemanea australis *exhibited high essential nutrient contents (fat, carbohydrate, protein, and nitrogen content) as well as high micronutrient content (Zn and Cu). The species was expensive and just three vendors were involved in its trade. The carbohydrate content was recorded high in the roots of *Nelumbo nucifera *and *Colocasia esculenta*. These species also showed high nutritional value. The wild edible plant resources formed an important part of local diets in mountain areas that also contributed significantly in nutritional balance among tribal communities [35,36].

The wetlands comprised an integral part of the indigenous socio-ecological system that has strong links with the traditional ecological knowledge available within the communities. In this study nearly 75% of wetland species showed ethnomedicinal properties. A study on Apatany Tribe of Northeast in Arunachal Pradesh highlighted 173 species having ethnomedicinal importance [37]. Even though the wetland resources provide good scope for revenue generation to the tribal communities, their longevity was under threat as traditional cultures have been eroding fast in recent times. Traditional culture was having sustaining harvesting practices. The major share of wetland plant collection used for home consumption. Most of the species were herbaceous and their frequent harvesting often resulted in overexploitation. Moreover, due to shrinkage of wetlands the availability of plant resources has been registering a declining trend during recent years. The availability of 45% species had decreased significantly over the years while 30% species demanding some kind of conservation measures in the investigated area. Frequent and over exploitation of species may lead a threat to their survival in near future as was observed for some species in Northeastern States [38,39]. The major threats on wetland areas were fishing, edible insect collection and the conversion of marginal land of wetlands to paddy cultivation. The species that were imperiled due to diverse threats comprised *Lemanea australis, Neptunia oleracea, Alocasia cuculata, Euryale ferox, Nelumbo nucifera, Schoenoplectus lacustris, Zizania latifolia, Oenanthe javanica *and *Zizania latifolia*. Besides, *Alpinia nigra, Ammomum aromaticum, Ammomum *sp., *Enhydra fluctuans, Oenanthe javanica, Polygonum barbatum, Sagittaria sagittifolia *and *Trapa natans *also required due attention for conservation. Another high risk plant was *Lemanea australis *confined to small pocket at the confluence of two rivers. In remote rural settlements where vegetable cultivation was not practiced and market supplies not organized, local inhabitants depend on wild vegetables [34]. Therefore, domestication of selected species was highly warranted [35,36]. As the foremost mitigation measure the communities have started cultivation of selected species, viz. *Alpinia galanga, Euryale ferox, Nelumbo nucifera, Neptunia oleracea *and *Schoenoplectus lacustris*.

The threat to wetland plants was not only because of extensive extraction from wetlands but also due to construction of ring-bands for fishing, siltation from the surrounding uplands due to overland flow, conversion of marginal wetlands into paddy fields, development projects and urbanization. The tribal community view the traditional systems (including wetlands) as ones in which they see themselves as part of the cultural landscape [40]. The plants growing exclusively in the open water bodies, such as *Neptunia oleracea, Euryale ferox, Nelumbo nucifera*, were affected most. At present *Neptunia oleracea *and *Euryale ferox *were not at all found in wild habitats. Development of agro-techniques and cultivation protocols were desired for species under high consumption and extraction pressures, and market demands. Rotational harvesting based on scientific studies on regeneration capacity and yield aspects of respective species can lead to develop proper harvesting regimes. Based on the community matrix ranking *Alocasia cuculata, Euryale ferox, Lemanea australis, Nelumbo nucifera, Neptunia oleracea, Schoenoplectus lacustris *and *Zizania latifolia *demanded immediate conservation measures as these species were of high demand in household use and trade. Management of species in the wild habitats also desired high awareness conservation education among users and collectors.

## Conclusions

The diverse use of wetlands plants for food, medicine and other socio-cultural purposes by the ethnic communities of Manipur revealed high dependence on these resources with as many as 51 plant species being collected, which also comprised 38 medicinal plants. 31 wetland species were sold in local markets thus fetched good income to rural communities. The most commonly traded wetland plants were *Euryale ferox, Colocasia esculenta, Nelumbo nucifera, Oenanthe javanica*, etc. The edible wetland species also form a good source of nutrients in local diets. *Lemanea australis, Colocasia esculenta, Zizania latifolia, Nelumbo nucifera *and *Polygonum barbatum *were most preferred for their nutrient contents. *Alocasia cuculata, Euryale ferox, Nelumbo nucifera, Neptunia oleracea, Schoenoplectus lacustris *and *Zizania latifolia *desired immediate conservation attention in view of their high exploitation. Based on communities' view points on uses, market demand, trade and conservation concerns, *Alocasia cuculata, Euryale ferox, Nelumbo nucifera, Neptunia oleracea *and *Zizania latifolia *were most significant species of the wetland areas of Manipur state.

For self-reliant development of wetland areas there was a need to strengthen community-wetland linkages by opting a conservation as well as livelihood development approach. The wetlands of the region can provide high income generating opportunities to local communities. It is suggested that a strong participatory approach is required for sustainable management of wetland area. To achieve the said goal, the community need be organized for adopting sustainable harvest protocols for all wetland species and needful training be imparted to them. Furthermore, proper value chain development for marketing and value-addition for selected wetland plants can bring good income to the communities. Most of the wetland plants have low self-life therefore improving keeping quality and developing by-products can help to increase income from them. Also, the most preferred wetland edible species can be domesticated in farmers' fields after developing proper agro-techniques for them, which will certainly help to reduce pressures on these species in wetland areas. Some medicinal plants may also be used to develop into modern medicines.

In recent times the wetland of the region faced an increasing threat because of construction of ring-bands for fishing, siltation from the surrounding uplands due to overland flow, conversion of marginal wetlands into paddy fields, development projects and urbanization of wild areas. Eco-restoration of wetland areas and conservation education to communities along with and opening of income generating avenues as proposed above along with other promotional activities, such as ecotourism, etc. would help in conserving these valuable resources in long-run. Therefore a detailed and comprehensive management strategy, based on cultural, ecological and economic principles need to be devised for each of the wetland area for their sustainable management.

## Competing interests

The authors declare that they have no competing interests.

## Authors' contributions

**AJ **carried out purposive sampling for wetland plants with relation to community dependence for subsistence and commercial needs and organised interview schedules. **MS **carried out nutrient analysis of commonly used and marketed wetland species. **SR, RK **and **PBK **participated in market surveys at Imphal, Bishenpur, and Thoubal markets, respectively. They also prepared vouchers specimen of all wetland species into the herbarium and identified them to species level. **HBS **collected information on wetland medicinal plants, their use pattern and processing. He conceived the study and participated in its design and coordination. **RCS **participated in the design of the study, performed the statistical analysis and compiled the information in the manuscript. All authors read and approved the final manuscript.
